# β1-integrin via NF-κB signaling is essential for acquisition of invasiveness in a model of radiation treated *in situ *breast cancer

**DOI:** 10.1186/bcr3454

**Published:** 2013-07-25

**Authors:** Jin-Min Nam, Kazi M Ahmed, Sylvain Costes, Hui Zhang, Yasuhito Onodera, Adam B Olshen, Kanako C Hatanaka, Rumiko Kinoshita, Masayori Ishikawa, Hisataka Sabe, Hiroki Shirato, Catherine C Park

**Affiliations:** 1 Department of Radiation Medicine, Hokkaido University Graduate School of Medicine, North-15, West-7, Sapporo, Hokkaido, 060-8638, Japan; 2 Life Sciences Division, Ernest Orlando Lawrence Berkeley National Laboratory, 1 Cyclotron Road, Berkeley, 94720, USA; 3 Department of Molecular Biology, Hokkaido University Graduate School of Medicine, North-15, West-7, Sapporo, Hokkaido, 060-8638, Japan; 4 Department of Epidemiology and Biostatistics, University of California San Francisco, 1450 3rd Street San Francisco, CA, 94158, USA; 5 Department of Surgical Pathology, Hokkaido University Hospital, North-15, West-7, Sapporo, Hokkaido, 060-8638, Japan; 6 Department of Medical Physics and Engineering, Hokkaido University Graduate School of Medicine, North-15, West-7, Sapporo, Hokkaido, 060-8638, Japan; 7 Department of Radiation Oncology, University of California San Francisco, California Comprehensive Cancer Center, 1600 Divisadero Street H1031, San Francisco, CA 94143-1708, USA

**Keywords:** ductal carcinoma *in situ*, DCIS, integrin, ionizing radiation

## Abstract

**Introduction:**

Ductal carcinoma *in situ *(DCIS) is characterized by non-invasive cancerous cell growth within the breast ducts. Although radiotherapy is commonly used in the treatment of DCIS, the effect and molecular mechanism of ionizing radiation (IR) on DCIS are not well understood, and invasive recurrence following radiotherapy remains a significant clinical problem. This study investigated the effects of IR on a clinically relevant model of Akt-driven DCIS and identified possible molecular mechanisms underlying invasive progression in surviving cells.

**Methods:**

We measured the level of phosphorylated-Akt (p-Akt) in a cohort of human DCIS specimens by immunohistochemistry (IHC) and correlated it with recurrence risk. To model human DCIS, we used Akt overexpressing human mammary epithelial cells (MCF10A-Akt) which, in three-dimensional laminin-rich extracellular matrix (lrECM) and *in vivo*, form organotypic DCIS-like lesions with lumina expanded by pleiomorphic cells contained within an intact basement membrane. In a population of cells that survived significant IR doses in three-dimensional lrECM, a malignant phenotype emerged creating a model for invasive recurrence.

**Results:**

P-Akt was up-regulated in clinical DCIS specimens and was associated with recurrent disease. MCF10A-Akt cells that formed DCIS-like structures in three-dimensional lrECM showed significant apoptosis after IR, preferentially in the luminal compartment. Strikingly, when cells that survived IR were repropagated in three-dimensional lrECM, a malignant phenotype emerged, characterized by invasive activity, up-regulation of fibronectin, α5β1-integrin, matrix metalloproteinase-9 (MMP-9) and loss of E-cadherin. In addition, IR induced nuclear translocation and binding of nuclear factor-kappa B (NF-κB) to the β1-integrin promoter region, associated with up-regulation of α5β1-integrins. Inhibition of NF-κB or β1-integrin signaling abrogated emergence of the invasive activity.

**Conclusions:**

P-Akt is up-regulated in some human DCIS lesions and is possibly associated with recurrence. MCF10A-Akt cells form organotypic DCIS-like lesions in three-dimensional lrECM and *in vivo*, and are a plausible model for some forms of human DCIS. A population of Akt-driven DCIS-like spheroids that survive IR progresses to an invasive phenotype in three-dimensional lrECM mediated by β1-integrin and NF-κB signaling.

## Introduction

Ductal carcinoma *in situ *(DCIS) is comprised of cancerous cells that are contained within the milk duct and separated from the stroma by a basement membrane and is associated with risk for developing invasive cancer [1]. With the advent of screening mammography, DCIS represents approximately 20% of all new cases of breast cancer diagnosed in the United States annually. Lumpectomy followed by radiation therapy (RT) is the most common treatment for DCIS, the efficacy of which is supported by randomized trials and meta-analysis demonstrating a reduction in the risk for local recurrence by approximately 50% [2,3]. Although the primary goal of therapy is to prevent invasive recurrence, 50% of all local recurrences after RT are invasive cancer [2]. Relevant experimental models to investigate molecular effects of ionizing radiation (IR) on invasive recurrence, however, have not been established.

Although structurally separated from the stromal breast tissue by a basement membrane, DCIS lesions in humans have the ability to elicit stromal remodeling of the extracellular matrix (ECM); indeed, stromal neoangiogenesis and fibronectin (FN) deposition have been well documented [4-6]. β1-integrins are critical mediators of normal cell-ECM interactions and have been shown to play a multifaceted role in malignant progression [7]. Others and we have shown that IR results in up-regulation of β1-integrins in invasive breast cancer, leading to increased cell survival [8-10].

Recently, we turned our attention to other mediators of the acute phase response to IR. It is known that nuclear factor-kappa B (NF-κB) is a pleiotropic regulator of many genes involved in inflammation, growth regulation and apoptosis, and IR [11-13]. In fact, several reports place integrins upstream of NF-κB [14-16]. We recently showed that upon IR exposure in breast cancer cells, NF-κB binds directly to the β1-integrin promoter region, resulting in increased β1-integrin transcripts and radioresistance [17]. Here, we verified the importance of NF-κB regulation of β1-integrin post-IR in the context of Akt-driven progression.

In the present study, we show that phosphorylated-Akt (p-Akt) is up-regulated in clinical DCIS specimens and is associated with recurrent disease. To investigate the possible molecular mechanisms of IR on DCIS, we used active Akt-overexpressing human mammary epithelial cells, MCF10A-Akt, in three-dimensional lrECM cultures as a model of DCIS, which we validated *in vivo*. When propagated in three-dimensional lrECM, the MCF10A-Akt cells recapitulate an organotypic duct-like structure that retains basal polarity, but with lumina expanded by pleiomorphic cells resembling human DCIS [18]. DCIS-like MCF10A-Akt structures in three-dimensional lrECM show a significant increase in apoptosis in response to IR, preferentially in the luminal compartment. To determine whether surviving cells remained viable after IR, we selected and repropagated them in three-dimensional lrECM. Strikingly, we observed the emergence of a malignant phenotype in a sub-population of survivors, with increased β1-integrin expression, matrix metalloproteinase-9 (MMP-9) and invasive activity. In addition, among the malignant population, IR induced nuclear translocation and binding of NF-κB p65 to the β1-integrin promoter region, associated with up-regulation of β1-integrins. Inhibition of NF-κB translocation to the nucleus or inhibition of β1-integrin signaling abrogated the emergence of the invasive phenotype. These results indicate that regulation of β1-integrin signaling via NF-κB may play an important role in the emergence of invasive disease after radiation treatment of Akt-driven DCIS-like lesions.

## Methods

### Tissue specimens

Clinical specimens were obtained from 24 patients with pure DCIS, who were treated at the Hokkaido University Hospital from 1998 to 2008. Patients underwent breast-conserving surgery followed by external beam fractionated radiotherapy to the whole breast. Among the 24 patients, five had ipsilateral invasive breast tumor recurrence within five years. This study was approved by the Institutional Review Board of Hokkaido University Hospital (010-0203). The requirement for written consent was waived by our institutional board according to the Ethical Guidelines for Clinical Studies of the Japanese Ministry of Health, Labor and Welfare.

### Immunohistochemistry and pathologic scoring of human DCIS tissues

Immunohistochemistry (IHC) of human DCIS tissues was performed on 4 μm-thick formalin-fixed paraffin-embedded serial sections. Immunohistochemical staining of p-Akt was performed by using the CSAII Biotin-free Tyramide Signal Amplification System (DAKO, Tokyo, Japan) according to the manufacturer's protocol. Each slide was deparaffinized in xylene and dehydrated through graded alcohols, and processed for antigen retrieval by ethylenediaminetetraacetic acid (EDTA) (pH 9.0) at 95°C for 40 minutes. Endogenous peroxidase was blocked by 3% hydrogen peroxidase at room temperature for 10 minutes and then blocked by serum-free protein in buffer for 10 minutes. Primary antibody against p-Akt (1:50, Cell Signaling Technology, Danvers, MA, USA) was incubated overnight at 4°C. Slides were washed and then followed by sequential incubation for 15 minutes with anti-rabbit immunoglobulins-horseradish peroxidase (HRP) (1:200), fluorescyl-tyramide hydrogen peroxide and anti-fluorescein-HRP. For β1-integrin staining, EnVision™ system (DAKO) was used. After deparaffinization, the slides were treated with antigen retrieval reagent (pH 9.0) at 95°C for 40 minutes. Slides were washed and incubated in 3% H_2_O_2 _and then blocked. After rinsing, the sections were incubated with primary antibody against β1-integrin (1:150) overnight at 4°C. Antibody detection was performed using the EnVision™ system. The color was developed with 3, 3'-diaminobenzidine tetrahydrochloride (DAB)/hydrogen peroxide. Each slide was counterstained with hematoxylin. Blinded samples were reviewed by a pathologist and scored for nuclear grade and Van Nuys classification. IHC was scored based on the intensity of signal (0 = none, 1 = light, 2 = moderate, 3 = heavy) and the percentage of positive cells (0 = <10%, 1 = 10% to 25%, 2 = 25% to 50%, 3 = >50%). All variables were made binary before analysis. All patients had at least five years of follow-up, and thus, we estimated five-year recurrence-free survival rates. For the other variables, if they were not already binary, such as estrogen receptor (ER) status, a threshold was used that led to a balance in the counts of the two groups. Associations between variables were assessed using Fisher's exact test. Tests with *P*-values less than 0.05 were considered significant.

### *In vivo *study

To mimic human DCIS, intraductal transplantation was performed as described previously [19]. Adult 9-week old virgin female SCID mice were anesthetized, a Y-incision was made on the abdomen, and then the nipple of the 4th inguinal mammary gland was identified and transected. Forty thousand MCF10A-Akt cells in 2 to 4 μl medium containing 0.1% trypan blue were injected *via *the cleaved nipple of mouse mammary ducts. A 50 μl capacity Hamilton syringe with a 30-gauge blunt-ended 1/2-inch needle was used to inject the cells. After the injection, the skin was closed with wound clips. A sterile tamoxifen pellet (Innovative Research of America, Sarasota, FL, USA) was inserted subcutaneously. At the end of the experiments, mammary glands were harvested, and paraffin or frozen blocks were made. All animal experimental procedures were approved by the Lawrence Berkeley National Laboratory Animal Welfare and Safety Committee.

### Immunohistochemistry and immunofluorescence on *in vivo *tumor sections

IHC of thin sections of mouse mammary gland tissues containing human-like DCIS lesions was performed using standard avidin-biotin-peroxidase (ABC) methods. Each slide was baked at 60°C for 30 minutes, dewaxed in xylene and rehydrated through graded alcohols. For β1-integrin staining, slides were incubated with proteinase K at 37°C for 10 minutes. After washing, slides were microwaved for 10 minutes in 10 mmol/L sodium citrate (pH 6.0) and cooled for 30 minutes. Each section was blocked with 10% normal rabbit serum for β1-integrin or normal goat serum for p-Akt and cleaved caspase-3 staining for 15 minutes. Primary antibody against β1-integrin (1:50, Millipore, Billerica, MA, USA) was incubated for one hour. Primary antibody against p-Akt (1:50, Cell Signaling Technologies) or cleaved caspase-3 (1:300, Cell Signaling Technologies) was incubated overnight at 4°C. Antibody detection was performed using an avidin-biotinylated enzyme. The coloring reaction was performed with DAB. Each section was counterstained with hematoxylin. For immunofluorescence (IF) staining of Ki-67, each slide was baked and deparaffinized in xylene, and rehydrated through a graded series of ethanol and then microwaved for 10 minutes in 10 mmol/L sodium citrate (pH 6.0). After cooling for 20 minutes, the sections were blocked by 10% goat serum for 10 minutes. Primary antibody against Ki-67 (1:500, Vector laboratories, Burlingame, CA, USA) was incubated overnight at 4°C. Slides were washed and incubated with secondary antibody for 40 minutes. Nuclei were counter stained with 4', 6-diamino-2-phenylindole (DAPI; Sigma-Aldrich, St. Louis, MO, USA). The images of IHC and IF were acquired by using a Zeiss Axioskop inverted microscope.

### Cell culture

MCF10A cells expressing ER-Akt (MCF10A-Akt), a kind gift from Dr. Jayanta Debnath (University of California in San Francisco, UCSF) were cultured in (D)MEM/F12 supplemented with 5% horse serum (Life technologies, Carlsbad, CA, USA); 20 ng/ml epidermal growth factor (EGF; Roche, Basel, Schweiz); 10 μg/ml insulin (Sigma-Aldrich); 0.5 μg/ml hydrocortisone (Sigma-Aldrich); 100 ng/ml cholera toxin (Sigma-Aldrich) and penicillin-streptomycin (Life technologies) [20]. For ER-Akt activation, 4-hydroxytamoxifen (4-HT; Sigma-Aldrich) was added to the culture medium on day two of three-dimensional culture, and EtOH was used as a control.

### Three-dimensional lrECM culture

For three-dimensional lrECM cultures, MCF10A-Akt cells were plated on top of commercially available growth factor-reduced Basement Membrane Extract (BD Biosciences, San Jose, CA, USA) as described previously [21]. MCF10A-Akt cells were cultured on three-dimensional lrECM for 12 days in the presence of EtOH or 1 μM 4HT. At Day 12, cultures were exposed to Sham, 2 or 8 Gy X-ray and harvested on Day 15. For the recurrence model, the colonies were isolated and dissociated from three-dimensional lrECM to make single cells using 0.05% trypsin-EDTA, and expanded on two-dimensional plastic on Day 15. Single cells were re-plated on top of three-dimensional lrECM and propagated for 12 additional days.

### Lysis from three-dimensional lrECM

To release cells from three-dimensional lrECM, cultures were first treated with ice-cold 5 mM EDTA/PBS on ice and then cells were lysed in 1% radioimmunoprecipitation assay (RIPA) buffer (1% Noidet P-40, 150 mM NaCl, 50 mM Tris-HCl (pH 7.4), 5 mM EDTA, 1% Na-deoxychorate, 0.1% SDS, 1 mM Na_3_VO_4_, 10 μM Na_2_MoO_4_, and protease inhibitor cocktail (Millipore)). After sonication, the solution was centrifuged and the supernatant was collected.

### Immunoblotting

Cell lysates were aliquoted onto Novex^® ^4-20% Tris-Glycine gels (Life technologies) in equal amounts and separated using 110 voltage current. Protein bands were transferred onto a polyvinylidene fluoride (PVDF) membrane, and blots were blocked with 5% skim milk/TBST (Tris-buffered saline Tween-20; 25mM Tris-HCl (pH7.4), 120 mM NaCl, 3 mM KCl and 0.1% Tween 20). Blots were probed with primary antibody (1:100 to 1:2,000 dilution in 3% skim milk/TBST) overnight at 4°C. After washing three times with TBST for 10 minutes, membranes were incubated with secondary antibody for one hour, then washed with TBST, and exposed by enhanced chemiluminescence.

### Immunostaining

Cells from three-dimensional cultures were fixed with 4% paraformaldehyde for 15 minutes, washed three times with glycine/PBS for 10 minutes and blocked with blocking buffer (10% goat serum (Life technologies), 1% Goat F(ab')2 anti-mouse antibody (Life technologies) in IF wash buffer (0.05% NaN_3_, 0.1% BSA, 0.2% Triton-X 100 and 0.05% Tween 20 in PBS)) for 1.5 hours in a humidified chamber. After removing blocking buffer, samples were incubated with primary antibody in blocking buffer overnight at 4°C and then washed three times with IF wash buffer, followed by incubating with Alexa Fluor 488-conjugated secondary antibody (Life technologies) for 40 minutes in a dark humidified chamber and then washed three times with IF wash buffer for 20 minutes. Nuclei were counterstained with DAPI, washed twice with PBS for 10 minutes and mounted. Images were acquired using a Zeiss Axioskop inverted microscope or a Nikon A1R confocal microscope system.

### Antibodies

The following antibodies were used: anti-β1-integrin, clone 18 (BD Biosciences); anti-β1-integrin, AIIB2 (Sierra Biosource, Morgan Hill, CA, USA); anti-α5-integrin (Millipore); anti-α6-integrin, NKI-GoH3 (Millipore); anti-FN, IST-4 (Sigma); anti-FN EDA+ (Abcam, Cambridge, England); anti-cleaved caspase-3 (Cell signaling Technology); anti-NF-κB p65 (Immuno-Biological Laboratories Co., Ltd., Gunma, Japan); anti-histone H1 (abcam) anti-β-actin (Sigma-Aldrich); ECL™ anti-mouse immunoglobulin G (IgG), HRP linked whole antibody (from sheep), NA931V (GE Healthcare, Buckinghamshire, UK); ECL™ anti-rabbit IgG, HRP linked whole antibody (from donkey), NA934V (GE Healthcare).

### Matrix degradation assay

Glass-bottomed dishes (MatTek, Ashland, MA, USA) were coated with DQ-gelatin from pig skin fluorescein conjugate (Invitrogen), which was subsequently cross-linked with 0.5% glutaraldehyde on ice. Cells from three-dimensional lrECM cultures at day 30 were replated onto the coated dishes, incubated for 24 hours and then fixed with 4% paraformaldehyde for 15 minutes. The number of cells positive for matrix degradation was counted from 100 cells.

### Zymography

Gelatin gels for the zymography assay were purchased from Invitrogen and handled according to the manufacturer's instructions. Cells were repropagated in three-dimensional lrECM cultures for five days and the medium was replaced by (D)MEM/F12 supplemented with additives (20 ng/ml EGF; 10 μg/ml insulin; 0.5 μg/ml hydrocortisone; 100 ng/ml cholera toxin). After 24 hours, the medium absent of serum was collected and denatured in SDS buffer under non-reducing conditions, and run on a Novex^® ^Zymogram gel. After electrophoresis, the gel was incubated in Zymogram renaturing buffer and then equilibrated in Zymogram developing buffer.

### Matrigel chemoinvasion assay

The matrigel chemoinvasion assay was performed using Biocoat Matrigel invasion chambers (BD Biosciences) as described previously [22]. Briefly, 5 × 10^4 ^cells were seeded onto the upper wells of 24-well chambers in the absence of serum, and the lower wells were filled with culture medium. After incubation for 18 hours, cells that migrated out onto the lower surface of the membranes were fixed with 4% paraformaldehyde at room temperature for 15 minutes. These cells were stained with 1% crystal violet and analyzed using AxioCam (Carl Zeiss, Jena, Germany). Data were collected from three independent experiments.

### Apoptosis assay

Apoptosis was detected using terminal deoxynucleotidyl transferase-mediated dUTP nick end labeling (TUNEL) as described previously [10]. Samples from three-dimensional lrECM were fixed with 4% paraformaldehyde and permeabilized in 0.1% Triton X-100 in 0.1% sodium citrate. After washing with PBS, cells were incubated in TUNEL reaction mixture (In Situ Cell Death Detection Kit, Roche) at 37°C for 60 minutes.

### Image acquisition, processing and analysis

Cells were viewed and imaged using a Zeiss Axio Observer Z1 automated microscope (Carl Zeiss). Images were acquired using a high NA Zeiss plan-apochromat 20X (NA of 0.8) and a sensitive scientific-grade EM-CCD camera (Axiocam, Carl Zeiss). In order to acquire data for a large number of colonies, minimize acquisition time and simplify analysis, the centers of individual acini were marked with the 'Mark and Find' function of Axiovision and images for all acini were acquired at once. In order to compare immunostaining intensity, for a given antibody, all images across treatments were acquired with the same exposure time, making sure images were not saturated. Each treatment group contained 100 to 300 acini in duplicate experiments. Image processing was performed under Matlab (MathWorks Inc, Natick, MA, USA) and DIP image (image processing toolbox for Matlab, Delft University of Technology, The Netherlands). Statistical tests were done with the Statistics module of Matlab. Results of three-dimensional studies are expressed as mean ± SE. Data were analyzed by Student's *t *test. *P *values of < 0.05 were considered significant. Cells positive for cleaved caspase-3 were identified by setting a constant threshold across all treatments above which pixels were considered positive if they were also overlapping with the nuclear DAPI area. The percent of positive cells was computed by dividing the positive area with the nuclear DAPI area. All imaging parameters and methods used in this manuscript have recently been described [23].

## Results

### Phosphorylated-Akt and β1-integrin are up-regulated in human DCIS specimens and correlate with disease recurrence

**Figure 1:**
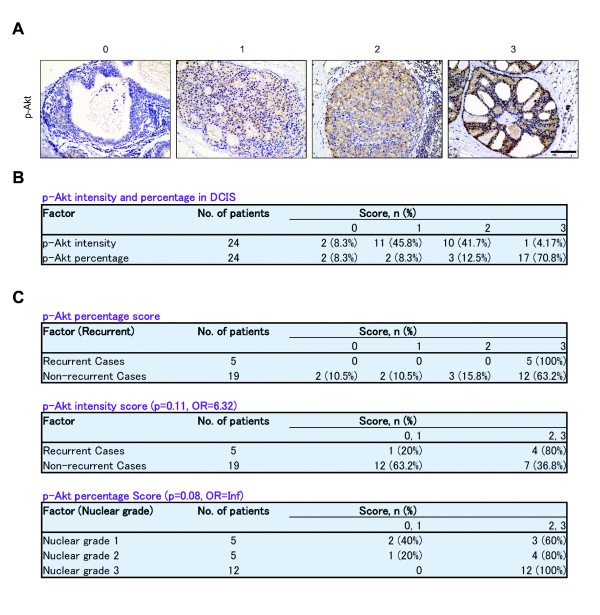
**Phosphorylated-Akt is up-regulated in clinical DCIS specimens**. (**A**) IHC of p-Akt in human DCIS specimens. Formalin-fixed, paraffin-embedded DCIS sections from 24 patients were stained with p-Akt. Phosphorylated-Akt intensity score: 0 = none, 1 = light, 2 = moderate, 3 = heavy. Bar = 100 μm. (**B**) Intensity and percentage expression pattern for p-Akt in human DCIS specimens. Phosho-Akt percentage score: 0 = <10%, 1 = 10% to 25%, 2 = 25% to 50%, 3 = >50%. (**C**) Up-regulation of p-Akt is associated with recurrent disease in human DCIS. DCIS, ductal carcinoma *in situ*; IHC, immunohistochemistry.

Oncogenic Akt activation has been implicated in breast cancer progression and is also up-regulated in clinical human DCIS tissue specimens [24]. To investigate the clinical importance of p-Akt and β1-integrin levels in human DCIS, we asked whether their expression level was correlated with local recurrence after breast conserving surgery and RT in a limited cohort of patients. First, we confirmed that both p-Akt and β1-integrin were indeed present in human DCIS specimens (Figure 1A and Additional file 1A). Among the 24 patients, 22 (91.7%) scored positively (1 to 3 versus 0) for p-Akt intensity and percentage. Importantly, 17 (70.8%) cases had a percentage score of 3, or the highest level of expression (Figure 1B). We then analyzed p-Akt staining for association with local recurrence within five years (Figure 1C). Among the five recurrent cases, all five (100%) had a percentage score of 3 compared with the non-recurrent cases (63.2%). For p-Akt intensity, moderate to high expression (2 to 3 versus 0 to 1) was strongly associated with recurrent cases (80%), whereas 0 to 1 scores are dominant in non-recurrent cases (63.2%) (Figure 1C; odds ratio (OR) = 6.32, *P *= 0.11, Fisher's exact test). Since nuclear grade is known to be one of the most important clinical factors associated with disease recurrence in DCIS, we also tested for the association between p-Akt expression and nuclear grade. Among the 22 scorable cases, 12 cases are nuclear grade 3, and all 12 had a moderate to high percentage (2 or 3) of p-Akt.

We also examined β1-integrin expression, comparing recurrent versus non-recurrent cases [see Additional file 1]. We found that a high percentage of β1-integrin expression is associated with recurrent cases compared with non-recurrent cases (40% versus 5.3%, OR = 10.8, *P *= 0.09). These results are consistent with our previous study in early stage invasive breast cancer where we showed that β1-integrin expression was associated with more aggressive disease and significantly reduced overall and disease-free survival [25]. These data suggest that p-Akt and β1-integrin are important in identifying aggressive forms of DCIS that is more likely to recur following treatment.

### Akt-overexpressing MCF10A cells formed DCIS-like structures in three-dimensional lrECM cultures and *in vivo *

**Figure 2:**
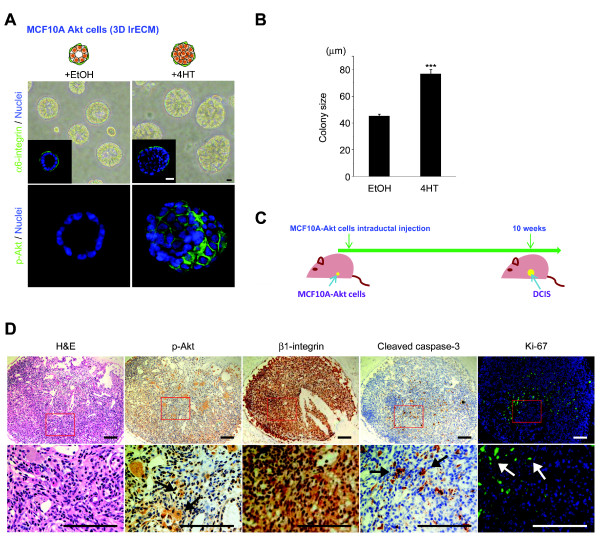
**Phosphorylated-Akt up-regulated MCF10A cells form DCIS-like structures in three-dimensional lrECM cultures and *in vivo***. (**A**) MCF10A cells form acinar-like structures with hollow lumina when propagated in three-dimensional lrECM. When p-Akt is overexpressed (MCF10A-Akt), the colonies are significantly larger with cells filling the lumina. Phase-contrast micrographs and IF images stained with α6-integrin or p-Akt are shown. Bar = 10 μm. (**B**) The average colony size is increased in MCF10A-Akt compared to MCF10A. (**C**) Experimental schema of *in vivo *study. The MCF10A-Akt cells were injected intraductally into the mouse mammary duct and subsequently generated DCIS-like lesions. (**D**) H & E, IHC (β1-integrin, p-Akt and cleaved caspase-3) and IF (Ki-67) staining of intraductal xenografts. H & E stained image from the xenograft is almost identical to clinical human DCIS. Bar = 100 μm. DCIS, ductal carcinoma *in situ*; IF, immunofluorescence; IHC, immunohistochemistry; lrECM, laminin-rich extracellular matrix.

We sought to investigate the effect of radiation and its underlying molecular mechanisms in DCIS. As human DCIS lesions were found to up-regulate p-Akt, we modeled DCIS by propagating MCF10A human mammary epithelial cells with inducible active ER-Akt [18,24]. Typically, MCF10A cells form a hollow lumen in three-dimensional lrECM [20], recapitulating normal *in vivo *ductal structures. However, cells induced to express activate Akt by adding 4-HT formed overgrown large colonies containing lumen filled with atypical appearing cells (Figure 2A and 2B), emulating premalignant *in situ *lesions. Notably, IF of α6-integrin indicates that the basal polarity is not disrupted, confirming the non-invasive structure (Figure 2A, inset).

To determine the validity of MCF10A-Akt cells as a model for human DCIS in three-dimensional lrECM, we recapitulated the DCIS-like structures *in vivo *(Figure 2C) using an established intraductal transplantation method [19]. The MCF10A-Akt cells were directly injected into the mammary duct of female SCID mice via a cleaved nipple and mice were monitored for up to 10 weeks. Using H & E staining from sections of harvested mammary glands, we detected intraductal tumors generated from MCF10A-Akt cells that represented human DCIS-like lesions (Figure 2D). Confirming that the molecular phenotype of these lesions emulated that observed in three-dimensional lrECM, co-overexpression of p-Akt and β1-integrin was detected by IHC (Figure 2D). The expression of cleaved caspase-3, an apoptosis marker, was negatively correlated with proliferation, measured by Ki-67 nuclear antigen, and negatively correlated with active Akt positive regions (Figure 2D). These results show that MCF10A-Akt cells form human DCIS-like structures *in vitro *in three-dimensional lrECM and *in vivo *that are proliferative and have high β1-integrin expression. Our *in vivo *data further validated the use of the MCF10A-Akt cells in the three-dimensional lrECM culture model as a surrogate for testing the effects of IR on human DCIS. Moreover, the *in vitro *system would allow us to further investigate the biology of recurrence following IR treatment of DCIS, which to date has not been adequately addressed.

### IR induces apoptosis in an Akt-overexpressing model of human DCIS in three-dimensional lrECM

**Figure 3:**
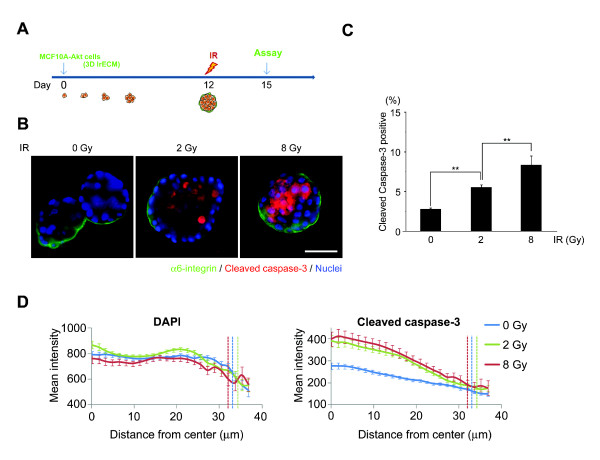
**IR induces apoptosis in an active Akt-overexpressing model of human DCIS in three-dimensional lrECM**. (**A**) Experimental schema. (**B**) IR-induced apoptosis was specifically observed in the luminal compartment of MCF10A-Akt structures. (Green = α6-integrin; red = cleaved caspase-3; blue = nuclei) Bar = 50 μm. (**C**) High content image analysis confirmed an increasing percentage of cells positive for cleaved caspase-3 with increasing IR doses. (*n *= 200 acini, **, *P *< 1E-7) (**D**) Concentric measurements of mean intensity of cleaved caspase-3 showed significantly higher signal in the lumina of irradiated acini, compared to unirradiated controls. Dashed lines indicated edge of the acini. DCIS, ductal carcinoma *in situ*; IR, ionizing radiation; lrECM, laminin-rich extracellular matrix.

Using the three-dimensional lrECM culture model, we next investigated the effect of IR on DCIS. To test whether radiation induces apoptosis in the DCIS model, we irradiated the three-dimensional cultured MCF10A-Akt cells at Day 12 and performed IF with anti-cleaved caspase-3 (Figure 3A). IR induced apoptosis of MCF10A-Akt cells in three-dimensional lrECM in a dose-dependent fashion, detected by cleaved caspase-3 (Figure 3B and Additional file 2). We further quantitated these results using high content image analysis which showed that cleaved caspase-3 positive cells increased with increasing radiation doses (Figure 3C). Interestingly, apoptotic cells were significantly increased in luminally located cells compared to basal cells that were in contact with ECM (Figure 3B and 3D).

### An invasive phenotype with high β1-integrin expression emerged from a sub-population of surviving cells post-IR in three-dimensional lrECM

**Figure 4:**
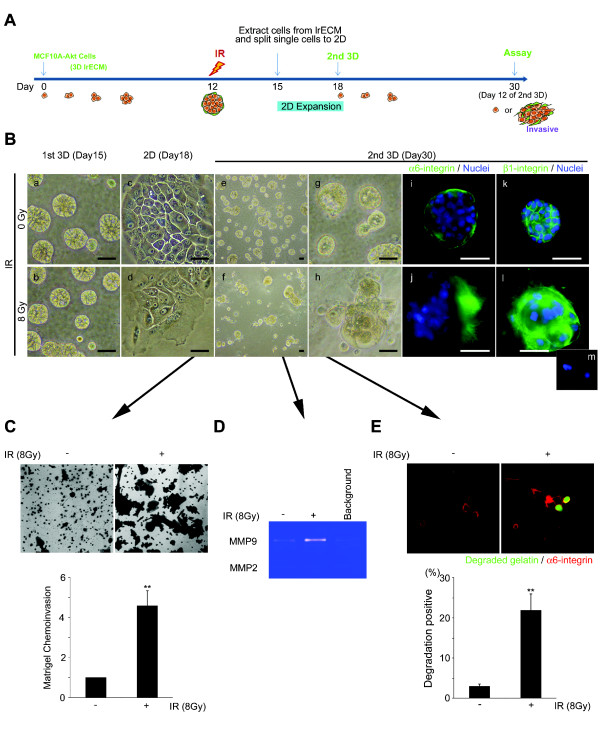
**An invasive phenotype emerged from a sub-population of cells surviving post-IR in three-dimensional lrECM**. (**A**) Experimental schema of the recurrence model. At Day 12, cultures were exposed to Sham or 8 Gy IR. On Day 15, the colonies were taken out of three-dimensional lrECM, dissociated to make single cells, and expanded on two dimensional. Single cells were re-plated on three-dimensional lrECM and propagated until Day 30 (12 additional days). (**B**) Phase-contrast micrographs show that a distinct phenotype emerged by Day 30 of culture. Bar = 50 μm. IF images show α6-integrin or β1-integrin (green). Bar = 50 μm. (**C**) Invasive activity of MCF10A-Akt cells post-IR was quantified using invasion chambers. Graphical representation of the invasive cell numbers were normalized with control, non-irradiated cultures (*n *= 3; **, *P *< 0.01). (**D**) Gelatin zymography shows that MMP-9 secretion was increased in culture medium of IR-treated MCF10A-Akt. (**E**) Matrix degradation activity was confirmed by fluorescently labeled DQ-gelatin matrix. Degraded gelatin is shown in green (22% ± 7 invasive cells versus 3% ± 1; *n *= 3; **, *P *< 0.01). DCIS, ductal carcinoma *in situ*; IF, immunofluorescence; IR, ionizing radiation; lrECM, laminin-rich extracellular matrix; MMP-9, matrix metalloproteinase-9.

Invasive recurrence remains a significant problem following breast-conserving surgery and radiation for DCIS. The nature of recurrence remains elusive, and there are currently no models to investigate this aspect of the disease. Thus, we sought to develop a model to investigate the viability of DCIS-like cells that survive significant doses of IR. MCF10A-Akt cells were cultured in three-dimensions for 12 days, followed by sham or 8 Gy IR (Figure 4A). After three days, the MCF10A-Akt cells were released from three-dimensions, dissociated to single cells, and then repropagated in three-dimensional lrECM (Figure 4A and 4B). Surprisingly, after 12 additional days of culture (or Day 30 of total number of days), we observed a subset of the culture population that exhibited an invasive phenotype (MCF10A-Akt-invasive) (Figure 4B, f, h). Alpha6-integrin showed a disruption in basal polarity with an increase in β1-integrin expression (Figure 4B, j, l). In contrast, the polarity of sham irradiated cells was retained on the second three-dimensional cultures (day 30, Figure 4B, e, g, i) similar to the first three-dimensional cultures (day 15, Figure 4B, a). Matrigel chemoinvasion was increased by 4.57-fold post-IR (Figure 4C), concomitant with an increase in MMP-9 in the conditioned medium of IR treated MCF10A-Akt-invasive cells (Figure 4D). Matrix degradation activity measured by DQ-gelatin matrix was increased by 22-fold post-IR (Figure 4E). Importantly, we also observed the emergence of similar invasive colonies post-IR using a similar MCF10A-NeoT cell model, validating this phenomenon [see Additional file 3].

### FN and α5β1-integrin are required for invasive progression in MCF10A-Akt cells post-IR

**Figure 5:**
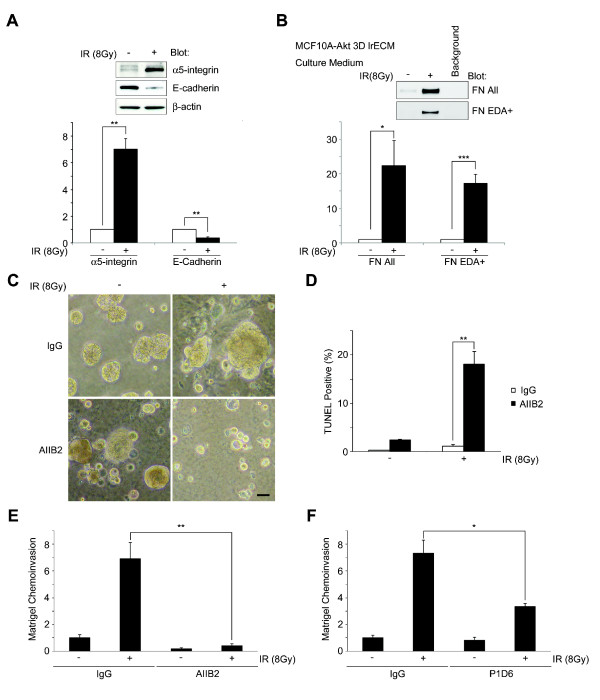
**β1-integrin signaling is targeted to suppress invasive recurrence post-IR in MCF10A-Akt cells in three-dimensional lrECM**. (**A**) On Day 30 cultures, up-regulated α5β1-integrin and down-regulated E-cadherin expression observed on the post-IR cultures. Columns, mean intensity (*n *= 3; **, *P *< 0.01). (**B**) Up-regulation of FN and EDA+FN was observed in culture medium of cells post-IR. Columns, mean intensity (*n *= 5; *, *P *< 0.05; ***, *P *< 0.001). (**C**) Phase-contrast images. IR-induced invasive phenotype was abrogated by AIIB2 compared to IgG. The antibodies were added from day 0 of the second three-dimensional cultures. Bar = 50 μm. (**D**) Apoptosis was measured by TUNEL-positive cells in AIIB2-treated cultures -/+ IR (mean = 18.1% ± 3.9, *P *< 0.01). (**E-F**) Matrigel chemoinvasion was significantly increased in the surviving cells post-IR, and inhibited by β1-integrin (AIIB2, five-fold, *P *< 0.01) or α5-integrin (P1D6, two-fold, *P *< 0.05) inhibitory antibodies. DCIS, ductal carcinoma *in situ*; FN, fibronectin; IgG, immunoglobulin G; IR, ionizing radiation; lrECM, laminin-rich extracellular matrix; TUNEL, terminal deoxynucleotidyl transferase-mediated dUTP nick end labeling.

We have previously shown that signaling downstream of α5β1-integrin and its ligand FN is important for breast cancer cell survival after radiation [10]. In addition to this, we have shown that the expression of α5β1-integrin, FN and EDA+FN, the FN variant expressing during embryogenesis and wound healing, is up-regulated in highly aggressive metastatic breast cells [10]. In the current study, we investigated whether α5β1-integrin and FN signaling is involved in the invasive tumor colonies post-IR on MCF10A-Akt in three-dimensional lrECM. At Day 30, the protein expression of α5-integrin was highly up-regulated and E-cadherin was down-regulated in the irradiated MCF10A-Akt cells in three-dimensional lrECM (Figure 5A). In addition, both total and EDA+FN were higher in the conditioned medium of irradiated cells versus control (Figure 5B).

Since β1-integrin was highly expressed in the invasive colonies and is a known driver of invasion, we tested whether inhibiting β1-integrin affected the ability of surviving cells post-IR to acquire invasive features. We found that β1-integrin inhibitory antibody, AIIB2, suppressed the progression of malignancy characterized by matrigel chemoinvasion activity and cancer cell survival after radiation treatment (Figure 5C, D and 5E). Beta1-integrin inhibition induced increased apoptosis (Figure 5D), and abrogated chemoinvasion activity (Figure 5E). We also found that α5β1-integrin inhibitory antibody could suppress the invasive activity (Figure 5F), indicating that α5β1-integrin heterodimer plays a specific role.

### NF-κB activation is involved in the emergence of the invasiveness in surviving MCF10A-Akt cells post-IR

**Figure 6:**
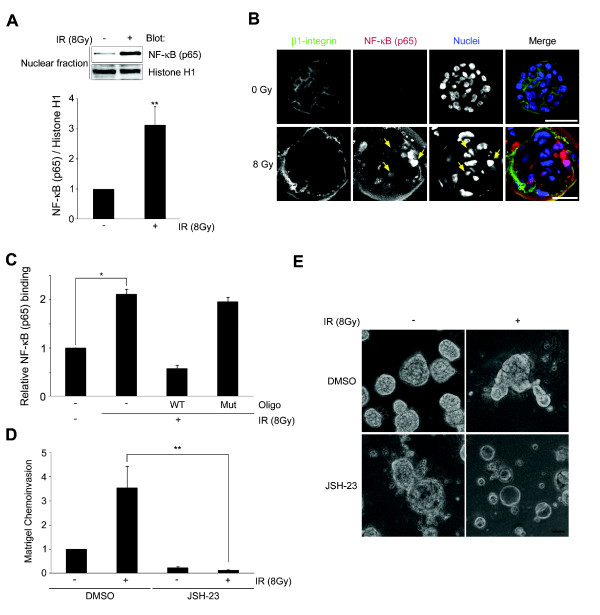
**Invasive phenotype emerged in a sub-population of irradiated MCF10A-Akt cells are associated with nuclear translocation of NF-κB**. (**A**) On Day 30 cultures, immunoblotting of nuclear fraction shows up-regulated nuclear translocation of NF-κB p65 in the 8 Gy IR cultures. The intensities of NF-κB were normalized with nuclear protein, Histone H1 (**, *P *< 0.01, *n *= 4). (**B**) IF images of confocal microscopy show β1-integrin (green), NF-κB (red) and nuclei (blue). Bar = 50 μm. (**C**) The binding of NF-κB to the β1-integrin promoter region is up-regulated in MCF10A-Akt cells that survived after exposure to radiation (WT, wild-type, Mt, mutated oligonucleotide; *n *= 3, *, *P *< 0.05). (**D-E**) The NF-κB inhibitor, JSH-23, was added from day 0 of the second three-dimensional cultures. IR-induced chemoinvasion activity, which was inhibited by NF-κB inhibitor, JSH-23. Bar = 50 μm. IF, immunofluorescence; IR, ionizing radiation; NF-κB, nuclear factor-kappaB; TUNEL, terminal deoxynucleotidyl transferase-mediated dUTP nick end labeling.

Among the possible molecular mechanisms involved in invasive recurrence downstream of FN and β1-integrin, our findings pointed to the potential role of NF-κB. NF-κB has been reported to induce pro-MMP9 expression downstream of FN and α5β1-integrin [26], and we recently showed its regulation of β1-integrin via binding to the β1-integrin promoter post-IR [17]. Thus, we hypothesized that increased FN-α5β1-integrin signaling via NF-κB/p65 activation could facilitate malignant progression post-IR on the three-dimensional culture model of DCIS. Figure 6A displays a three-fold induction of nuclear NF-κB/p65 by subcellular fractionation in MCF10A-Akt recurrent cells post-IR. IF also showed the nuclear localization of NF-κB/p65 in recurrent MCF10A-Akt cells post-IR (Figure 6B). In addition, the binding of NF-κB to the β1-integrin promoter region was significantly increased (Figure 6C). Treatment of MCF10A-Akt cells with NF-κB inhibitor, JSH-23, fully inhibited the IR-induced MCF10A-Akt-invasion activity (Figure 6D), suppressed formation of the invasive colonies (Figure 6E) and was associated with down-regulation of α5β1-integrin (data not shown). Taken together, these data suggest that feedback regulation of β1-integrin and NF-κB plays an important role in the emergence of invasive recurrence post-IR in our model of DCIS.

## Discussion

RT is commonly used in combination with breast conserving surgery for DCIS to reduce the risk of local recurrence. However, for many women, the risk of potentially life-threatening invasive recurrence remains a significant problem. In the present work, we used MCF10A-Akt cells in three-dimensional lrECM culture to model human DCIS and to investigate the molecular mechanisms involved in the IR effect on the emergence of invasive disease following treatment.

As others have previously reported [18], we also show that MCF10A-Akt cells form DCIS-like structures with an intact basement membrane and lumina expanded by pleiomorphic cells, reminiscent of DCIS *in vivo*. This model has clinical relevance since Akt has been reported to be overexpressed in human DCIS [24]. Furthermore, we examined p-Akt in several human DCIS cases that either recurred or had not recurred within five years after surgery and RT. Our data show that p-Akt is moderately to highly expressed in 17 of 24 (71%) of DCIS cases, and that five of five (100%) recurrent cases had a high percentage expression of p-Akt; and finally, among cases with the highest nuclear grade, p-Akt was moderately to highly expressed in all cases, providing further support for its role in disease progression. Using the active Akt-overexpressing DCIS model in three-dimensional lrECM, we found that, in response to IR, luminal cells preferentially undergo apoptosis compared to basally located cells. This is the first report, to our knowledge, of the effect of IR in a three-dimensional cell culture model of DCIS. It is well established that contact with ECM confers relative resistance to IR-induced death [27-30], and this has been corroborated in our own previous work [9]. However, the implications of the relative resistance of basally located cells that have more robust mechanotransduced signaling downstream of β1-integrin post-IR is not known.

To further investigate the fate of cells that remain viable post-IR, we selected for surviving MCF10A-Akt cells at Day 15 of culture. Strikingly, after expanding surviving cells in two dimensions, and repropagating these cells back into three-dimensional lrECM, we observed the growth of malignant colonies characterized by invasive activity by Day 30 of culture. This indicated that a subpopulation of viable cells progresses to invasive cancer; in addition, we believe that the disruption of the basal polarity of the DCIS and the expansion process from single DCIS cells were important in facilitating progression to invasive cancer in this model. In the clinical situation, our data implicate a role for β1-integrin signaling and NF-κB regulation in facilitating progression among even single cells that survive IR. In addition to the DCIS-Akt three-dimensional model, we confirmed the emergence of an invasive phenotype with up-regulation of FN/α5β1-integrin post-IR using a similar non-invasive MCF10A-NeoT cell model, indicating that the phenomenon was not dependent on a specific oncogenic driver.

The invasive colonies that emerged post-IR by Day 30 were characterized by diffuse α6-integrin distribution, high α5β1-integrin expression, increased MMP9, loss of E-cadherin, and the ability to invade. Beta1-integrin has been implicated in resistance to radiation treatment and mediating survival in breast cancer cells [9,10,29-31]. Thus, we hypothesized that β1-integrin could be targeted to eradicate the invasive colonies that emerged post-IR in our current DCIS model. As we expected, the β1-integrin inhibitory antibody could suppress the invasive phenotype and induced apoptosis of the cells, indicating the critical role for β1-integrins in the survival and progression of these cells that have the ability to invade after significant doses of IR.

We showed previously that the α5β1-integrin and FN were specifically up-regulated in breast cancer cells in three-dimensional lrECM and after IR [10]. Others have shown that FN and α5β1-integrin signaling via NF-κB induces MMP-9 expression in carcinoma cells [16,26,32]. MMP expression has been well correlated with invasive activity in breast cancer [33-36]. In the present study, we found that MMP-9 expression was associated with up-regulated expression of α5β1-integrin and total FN, as well as EDA+FN, and p-Akt in post-irradiated invasive cells. These results indicate a functional regulation between integrin/ECM signaling and proteinase mediated invasive activity, indicating that targeting FN and α5β1-integrin signaling via p-Akt could be an effective strategy to decrease the risk of invasive recurrence after the radiation treatment in DCIS patients.

Elevated NF-κB DNA binding activity has been demonstrated both in breast cancer cell lines and primary breast cancer tissues [37,38]. Constitutive activation of NF-κB contributes to malignant progression, radio- and chemo-resistance and increased metastasis of breast tumors [37,39]. In addition, we have demonstrated a forward loop-like regulation of β1-integrin by NF-κB post-IR, promoting survival in malignant breast cancer cells [17]. Maity *et al*. reported that the induction of MMP-9 expression and invasion of breast cancer cells are mediated by the activation of NF-κB via a FN/α5β1-integrin-dependent mechanism [26]. These reports and our findings indicate the possibility that IR-induced FN/α5β1-integrin expression is associated with NF-κB activity resulting in up-regulation of α5β1-integrin, MMP-9 secretion and invasiveness in MCF10A-Akt cells. Investigation of the detailed mechanism of the activation and function of NF-κB in these cells is currently underway.

## Conclusions

P-Akt is up-regulated and is associated with recurrent disease in a limited cohort of clinical DCIS specimens. Akt-overexpressing MCF10A-Akt cells form DCIS like structures in three-dimensional lrECM and *in vivo*, and when treated with IR, luminal cells preferentially undergo apoptosis. When cells surviving IR are selected and repropagated into three-dimensional lrECM, an invasive phenotype emerges, characterized by loss of basal polarity, loss of E-cadherin, ability to invade with increased MMP-9 activity, up-regulated FN, α5β1-integrin and nuclear translocation of NF-κB. Together, our results suggest that FN-β1-integrin via NF-κB feedback signaling iscritical mediators of invasive progression post-IR, and could be therapeutic targets to suppress invasive recurrence after radiation treatment of DCIS.

## Abbreviations

ABC: avidin-biotin-peroxidase; DAB: 3, 3'-diaminobenzidine tetrahydrochloride; DAPI: 4', 6-diamino-2-phenylindole; DCIS: ductal carcinoma *in situ*; (D)MEM: (Dulbecco's) modified Eagle's medium; EDTA: ethylenediaminetetraacetic acid; EGF: epidermal growth factor; FN: fibronectin; H & E, hematoxylin and eosin; HRP: horseradish peroxidase; 4-HT: 4-hydroxytamoxifen; IF: immunofluorescence; IgG: immunoglobulin G; IHC: immunohistochemistry; IR: ionizing radiation; MMP-9: matrix metalloproteinase-9; NF-κB: nuclear factor-kappa B; OR: odds ratio; p-Akt: phosphorylated-Akt; PVDF: polyvinylidene fluoride; RIPA: radioimmunoprecipitation assay; RT: radiation therapy; TBST: Tris-buffered saline Tween-20; three-dimensional lrECM, three-dimensional laminin-rich extracellular matrix; TUNEL: terminal deoxynucleotidyl transferase-mediated dUTP nick end labeling.

## Competing interests

CP is a co-founder of OncoSynergy, ltd. All other authors declare they have no competing interests.

## Authors' contributions

CP and JN developed the study concepts and designed the experiments; JN, KA, HZ and YO performed the experiments; SC performed the imaging analysis; KH conducted the pathological analyses; H. Shirato and H. Sabe supervised the clinical study. H. Sabe supervised data analysis; RK collected and organized clinical information of DCIS patients; AO performed statistical analyses; MI provided expertise in radiation physics. Administrative and material support was provided by MI, H. Sabe and H. Shirato; CP and JN prepared the manuscript with the assistance of YO, KA, HZ, SC, AO, RK, H. Shirato and H. Sabe. All authors read and approved the final manuscript.

## Supplementary Material

Additional file 1 **High percentage of β1-integrin expression is associated with recurrent cases compared with non-recurrent cases**. (**A**) IHC of β1-integrin in human DCIS specimens. Formalin-fixed, paraffin-embedded DCIS sections from 24 patients were stained with β1-integrin monoclonal antibody. All slides were counterstained with hematoxylin and representative image is shown for each intensity and percentage score. Scale bar, 100 μm. (**B**) Percentage expression pattern for β1-integrin in human DCIS specimens. Beta1-integrin percentage score: 0 = <10%, 1 = 10%-25%, 2 = 25%-50%, 3 = >50%. Click here for file

Additional file 2 **IR induces apoptosis in an Akt-overexpressing model of human DCIS in three-dimensional lrECM**. Western blot from total cell lysates showed increased expression of cleaved caspase-3 in 8 Gy irradiated MCF10A-Akt cells compared to 0 Gy. Equal amounts of protein were subjected to western blotting. The signals of cleaved caspase-3 were normalized with β-actin. Columns, mean intensity of western blot analysis (*n *= 3, *, *P *< 0.05). Click here for file

Additional file 3 **A phenotype of invasive recurrence with high α5β1-integrin expression emerged from a sub-population of surviving MCF10A-NeoT cells post-IR in three-dimensional lrECM**. (**A**) Experimental schema of the recurrence model. (**B**) Phase-contrast micrographs show that an invasive phenotype emerged by Day 30 of culture. (**C**) Up-regulated FN and α5β1-integrin protein level were observed on the 8 Gy IR compared to sham irradiated MCF10A-NeoT three-dimensional lrECM cultures. Click here for file
