# Supplementary material for: β1-integrin via NF-κB signaling is essential for acquisition of invasiveness in a model of radiation treated in situ breast cancer
Source: Breast Cancer Res. 2013 Jul 25;15(4):R60. doi: 10.1186/bcr3454 (PMC3978561; doi:10.1186/bcr3454)
Supplement: Additional file 1 — High percentage of β1-integrin expression is associated with recurrent cases compared with non-recurrent cases. (A) IHC of β1-integrin in human DCIS specimens. Formalin-fixed, paraffin-embedded DCIS sections from 24 patients were stained with β1-integrin monoclonal antibody. All slides were counterstained with hematoxylin and representative image is shown for each intensity and percentage score. Scale bar, 100 μm. (B) Percentage expression pattern for β1-integrin in human DCIS specimens. Beta1-integrin percentage score: 0 = <10%, 1 = 10%-25%, 2 = 25%-50%, 3 = >50%. [file bcr3454-S1.PDF]

Nam et al. Additional file 1

A

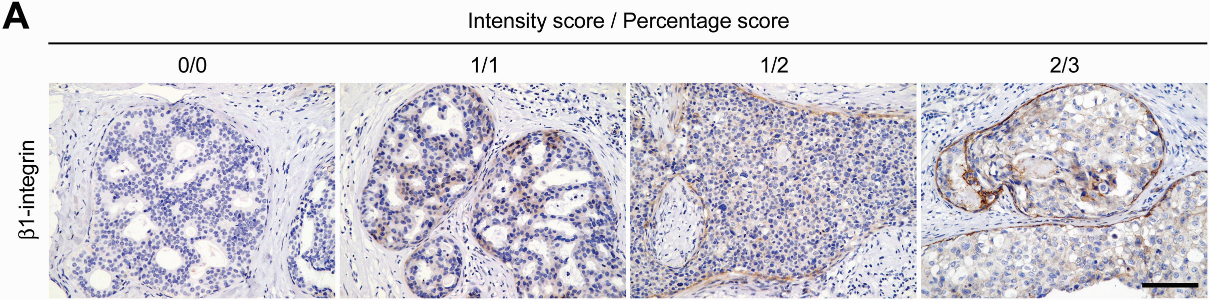

B

β1−integrin Percentage Score (p=0.09, OR=10.8)

Factor	No. of patients	Score, n (%)	
		0, 1	2, 3
Recurrent Cases	5	3 (60%)	2 (40%)
Non−recurrent Cases	19	18 (94.7%)	1 (5.3%)
