# Supplementary figures and images for: β1-integrin via NF-κB signaling is essential for acquisition of invasiveness in a model of radiation treated in situ breast cancer
Source: Breast Cancer Res. 2013 Jul 25;15(4):R60. doi: 10.1186/bcr3454 (PMC3978561; doi:10.1186/bcr3454)

# Nam et al. Additional file 2

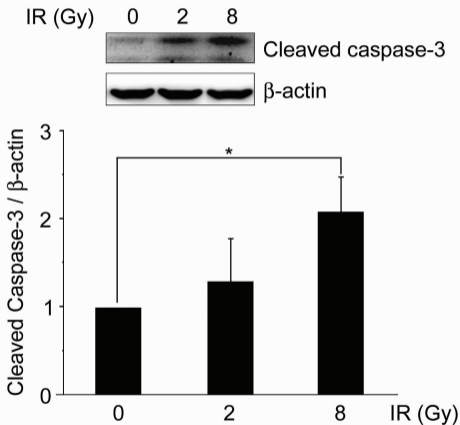

Supplement: Additional file 2 — IR induces apoptosis in an Akt-overexpressing model of human DCIS in three-dimensional lrECM. Western blot from total cell lysates showed increased expression of cleaved caspase-3 in 8 Gy irradiated MCF10A-Akt cells compared to 0 Gy. Equal amounts of protein were subjected to western blotting. The signals of cleaved caspase-3 were normalized with β-actin. Columns, mean intensity of western blot analysis (n = 3, *, P < 0.05). [file bcr3454-S2.PDF]

# Nam et al. Additional file 3

## A

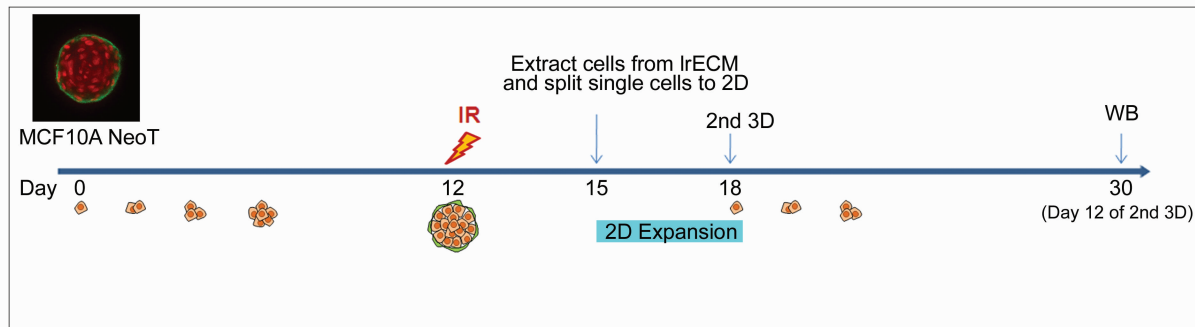

## B

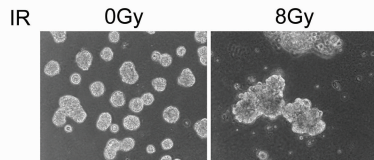

## C

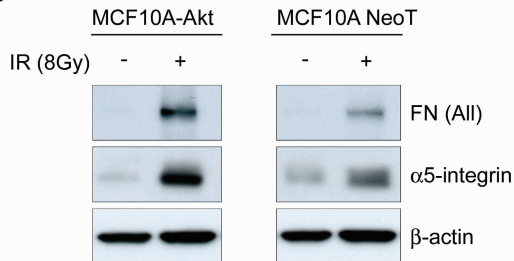

Supplement: Additional file 3 — A phenotype of invasive recurrence with high α5β1-integrin expression emerged from a sub-population of surviving MCF10A-NeoT cells post-IR in three-dimensional lrECM. (A) Experimental schema of the recurrence model. (B) Phase-contrast micrographs show that an invasive phenotype emerged by Day 30 of culture. (C) Up-regulated FN and α5β1-integrin protein level were observed on the 8 Gy IR compared to sham irradiated MCF10A-NeoT three-dimensional lrECM cultures. [file bcr3454-S3.PDF]
